# The Predictive Value of ^18^F-FDG PET/CT Radiomics in EGFR Gene Mutation of Lung Adenocarcinoma

**DOI:** 10.2174/0115734056428204251128060448

**Published:** 2026-01-13

**Authors:** Min Tang, Chunlei Zhao, Shengwei Fang

**Affiliations:** 1 Department of Radiology, Wuhan No. 1 Hospital, Wuhan, 430022, China; 2 Department of Nuclear Medicine, Hangzhou Cancer Hospital, Hangzhou, 310002, China

**Keywords:** Radiomics, ^18^F-FDG, PET/CT, Lung adenocarcinoma, Epidermal growth factor receptor, Radiomics model, Non-small cell lung cancer

## Abstract

**Introduction:**

This study aimed to evaluate the predictive value of radiomic features derived from ^18^F-FluoroDeoxyGlucose (FDG) PET/CT for Epidermal Growth Factor Receptor (EGFR) gene mutations in patients with lung adenocarcinoma.

**Methods:**

A retrospective analysis was conducted on 93 patients diagnosed with solitary lung adenocarcinoma who underwent ^18^F-FDG PET/ CT imaging and EGFR mutation results. The patients were divided into training (46 cases) and testing (47 cases) cohorts. Radiomic features were extracted from the primary tumor sites' PET and CT images. Feature selection was performed using the Mann-Whitney U test and least absolute shrinkage and selection operator (LASSO) regression. A radiomics score (Rad-score) was constructed, and combined models incorporating clinical factors and metabolic parameters were developed. Predictive performance was evaluated using receiver operating characteristic (ROC) curves, area under the curve (AUC), accuracy, and decision curve analysis (DCA).

**Results:**

The radiomics model achieved AUCs of 0.865 (95% CI: 0.747–0.983) and 0.737 (95% CI: 0.572–0.901) in the training and testing sets, respectively, with corresponding accuracies of 80.9% and 78.3%. The clinical model alone demonstrated inferior performance, with AUCs of 0.637 and 0.645. The combined model showed slightly improved AUCs (0.885 and 0.714) but did not significantly outperform the radiomics-only model (*P* > 0.05). DCA indicated greater clinical utility for the radiomics model across a wide range of threshold probabilities.

**Discussion:**

PET/CT-based radiomics research has also achieved good efficacy in predicting EGFR gene mutations. Compared with morphological imaging techniques, such as X-ray, ultrasound, and CT, ^18^F-FDG PET/CT imaging has the significant advantage of providing functional and metabolic information of lesions. Both radiomics and composite models could predict EGFR mutation status in lung adenocarcinoma patients, but the radiomics model showed slightly better clinical predictive efficacy than the composite model.

**Conclusion:**

The radiomics model and the combined model integrating Rad-score with clinical factors demonstrated comparable abilities in effectively predicting EGFR mutation status in patients with lung adenocarcinoma. These models could offer a non-invasive approach for identifying EGFR mutations.

## 1 INTROUDUCTION

Lung cancer remains a leading cause of cancer-related mortality worldwide, with non-small cell lung cancer (NSCLC) accounting for approximately 85% of cases. Lung adenocarcinoma is the most prevalent histological subtype [1, 2]. The identification of epidermal growth factor receptor (EGFR) mutations-found in approximately 50% of Asian patients with lung adenocarcinoma-has fundamentally transformed treatment paradigms [3]. The development of tyrosine kinase inhibitors (TKIs) has markedly improved survival outcomes for patients with EGFR-mutant lung adenocarcinoma (*e.g*., median overall survival of 30.8 months in a real-world setting [4, 5]. Therefore, accurate determination of EGFR status is essential for guiding therapeutic decisions [6].

Currently, tissue biopsy followed by molecular testing is the gold standard for detecting EGFR mutations. However, this approach is invasive, prone to sampling bias due to tumor heterogeneity, and not always feasible [7, 8]. Therefore, there is a compelling clinical need for reliable, non-invasive methods to predict EGFR mutation status.

^18^F-FluoroDeoxyGlucose(FDG) PET/CT, which provides both metabolic and anatomical information, plays a well-established role in the diagnosis, staging, and response assessment of lung cancer [9, 10]. While conventional metabolic parameters such as maximum standardized uptake value (SUVmax), metabolic tumor volume (MTV), and total lesion glycolysis (TLG) have been investigated for predicting EGFR mutations, their clinical utility remains limited, and results are inconsistent across studies, largely due to biological and technical variability [11-14]. Jiang *et al*. reviewed previous studies and identified that a low SUVmax of distant metastases was associated with the presence of EGFR mutations in advanced lung adenocarcinoma. Different cutoff values of SUVmax (ranging from 7.0 to 9.91) were established to achieve relatively high areas under the ROC curve, ranging from 0.557 to 0.75 [15].

Radiomics, a high-throughput method for extracting quantitative features from medical images, provides a powerful approach for decoding tumor phenotype and genotype [16]. Some radiomics research results have been applied in clinical practice, such as radiomics analysis of CT images to differentiate benign and malignant lung nodules. Yang *et al*. demonstrated that a CT-based radiomics signature successfully discriminated between EGFR-positive and EGFR-negative cases in the training cohort (area under the curve (AUC) = 0.831) and the validation cohort (AUC = 0.789) [17].

^18^F-FDG PET/CT radiomics adds metabolic information of lesions compared to traditional radiomics methods, and is expected to mine more texture features. The previous review article contextualized these advances by summarizing evidence that PET/CT radiomics consistently outperforms qualitative image assessment and single-modality analysis [18]. It emphasized that combining features from CT, which captures textural heterogeneity, and PET, which captures metabolic heterogeneity, provides complementary data that enhances the predictive power for determining EGFR status. In conclusion, these studies provide compelling evidence that ^18^F-FDG PET/CT radiomics is a highly accurate, non-invasive tool for predicting EGFR mutations. The findings demonstrate that integrating features from both CT and PET scans, incorporating clinical data, employing machine learning techniques, and analyzing specific tumor subregions can enhance predictive accuracy to AUC values exceeding 0.95, thereby advancing this methodology toward clinical application [18]. Li *et al*. leveraged the high resolution of thin-section CT alongside PET radiomics. Using a cohort of 361 patients, they extracted 1,842 features from both imaging modalities. Machine learning algorithms, specifically the Least Absolute Shrinkage and Selection Operator (LASSO) and Support Vector Machine (SVM), were employed for feature selection and model construction. The optimal model incorporated seven selected features. Their results were highly impressive, with the combined PET/CT radiomics model achieving an AUC of 0.986 in the training set and 0.952 in the validation set [19].

This study intends to extract metabolic and structural texture features of lesions from ^18^F-FDG PET/CT images, combined with clinical factors, to develop and validate a clinically practical model that can predict EGFR mutations in patients with lung adenocarcinoma, in order to provide a basis for guiding individualized molecular targeted therapy and improving the prognosis of patients.

## 2 MATERIALS AND METHODS

This study was conducted in accordance with the Declaration of Helsinki, as revised in 2013, and received approval from the Ethics Committee of Hangzhou Cancer Hospital (approval number HZCH-2023-RE-36). The data collected are anonymous; therefore, the requirement for informed consent was waived.

### 2.1 Patients

Clinical and imaging data were collected from 93 patients, which were retrospectively analyzed from January 2018 to March 2024. The inclusion criteria were as follows: a diagnosis of lung adenocarcinoma confirmed by surgical or puncture biopsy with subsequent EGFR gene detection; an ^18^F-FDG PET/CT scan conducted within one month prior to treatment; and no history of other malignancies. The exclusion criteria included: prior antitumor treatment before the PET/CT scan; poor image quality due to significant respiratory or motion artifacts, or unclear tumor boundaries that made it difficult to delineate the volume of interest (VOI); and incomplete clinical or imaging data.

### 2.2 EGFR Mutation Detection

Tumor tissue samples were obtained through either surgical resection or biopsy. The status of EGFR mutations was analyzed using the human EGFR gene mutation detection kit provided by Wuhan Friends Medical Technology Co., Ltd., China. Mutations in EGFR exons 18, 19, 20, and 21 were identified using the real-time PCR/amplification refractory mutation system (RT-PCR/ARMS). PCR analysis was performed on the PRISM 7500 system from Applied Biosystems, Inc. Experienced pathologists, each with over a decade of experience, interpreted and confirmed both the histological findings and the EGFR mutation results.

### 2.3 Image Acquisition

Prior to the scan, patients were required to fast for more than six hours and maintain a blood glucose level of less than 11.10 mmol/L. They were then administered ^18^F-FDG (produced by Shanghai Atom Science Pharmaceutical Co., Ltd., with a radiochemical purity exceeding 95%) at a dose of 3.7-7.4 MBq/kg. After a rest period of approximately 60 ± 10 minutes, patients underwent a PET/CT scan using the GE Discovery PET/CT 710. A low-dose CT scan was performed first, followed by a PET scan. The PET acquisition utilized a three-dimensional mode over six to eight beds, with each bed taking approximately two minutes. PET images, attenuated with CT data, were reconstructed using the ordered subset expectation maximization method (three iterations, 24 subsets, and a 4 mm full width at half maximum). To obtain more detailed morphological information, a breath-hold thin-section CT scan was performed immediately after the PET/CT scan. The acquisition parameters for this scan were set to a voltage of 120 kV, a current of 200 mA, a pitch of 1.15, a collimator width of 0.75 mm, a reconstruction slice thickness of 1.25 mm, and a matrix of 512 × 512.

### 2.4 ^18^F-FDG PET/CT Image Interpretation

Two experienced nuclear medicine physicians analyzed the PET images on the MedEx platform, employing the region of interest (ROI) technique to assess traditional metabolic parameters of the primary lung cancer lesion in three dimensions. These parameters include SUVmax, minimum standardized uptake value (SUVmin), mean standardized uptake value (SUVmean), peak standardized uptake value (SUVpeak), MTV, TLG, and the corresponding standardized uptake values of lean body mass (SUL): SULmax, SULmin, SULmean, and SULpeak. TLG is calculated as SUVmean × MTV.

### 2.5 Image Segmentation and Radiomics Feature Extraction

An experienced nuclear medicine physician manually delineated the primary tumor on each slice of the CT lung window images using 3D Slicer (v5.6.1). The resulting volume from these contours was defined as the VOI. A second senior physician then verified and adjusted this VOI. The maximum diameter of the tumor is defined as the largest diameter of the lesion observed on the lung window.

The VOI of the PET image was semi-automatically delineated using 40% of the SUVmax. For lesions near the mediastinum or bronchus, the CT image was used as a reference to ensure accurate boundary definition. Any discrepancies were resolved by consensus of inconsistency, a consensus was achieved through discussion. Prior to feature extraction, all images were resampled using the nearest neighbor interpolation method. This process ensured image isotropy and enhanced the reproducibility of the extracted features..

The spatial resampling intervals for all patients on the PET and CT images were standardized to 3 mm in the x, y, and z directions, followed by image standardization. PET/CT radiomic features were extracted using the Pyradiomics module in Python version 3.9.7, radiomic features were extracted from PET/CT images. The extracted features include the following categories: first-order features, which reflect the symmetry, uniformity, and local intensity distribution variations of the measured voxels, such as median, mean, minimum, maximum, standard deviation, skewness, and kurtosis; shape features, which quantitatively describe the geometric properties of the region of interest, including tumor surface area, volume, surface area-to-volume ratio, sphericity, compactness, and three-dimensional diameter, thereby reflecting the tumor's size and morphological characteristics in three dimensions; and second-order and higher-order texture features, which capture the spatial relationships between voxel intensities in medical images. These texture features represent perceivable or measurable spatial variations at the intensity level, considered as gray levels, and synthesize locally perceived image characteristics. Examples include the gray level co-occurrence matrix (GLCM), gray level run length matrix (GLRLM), gray level size zone matrix (GLSZM), neighboring gray tone difference matrix (NGTDM), and gray level dependence matrix (GLDM) [20-22].

In the image preprocessing stage, wavelet transform (WT) and Laplacian of Gaussian (LoG) filters were applied to process the original PET and CT images, as well as to extract radiomic features from both the original and filtered images [23]. WT is a mathematical framework for multi-resolution analysis. It decomposes an entire image into different frequency components (called “sub-bands”) at different scales. LoG is a single, fixed filter used primarily for feature detection (like edges and blobs) at a specific scale. The wavelet transform filtering consists of combinations of high-pass (H) and low-pass (L) filters applied to the PET/CT images in each dimension. Specifically, this includes Wavelet-LLH, Wavelet-LHL, Wavelet-LHH, Wavelet-HLL, Wavelet-HLH, Wavelet-HHL, Wavelet-LLL, and Wavelet-HHH.

### 2.6 Feature Selection and Model Construction

Considering the potential issues of multicollinearity and overfitting in high-dimensional feature analysis, this study employed the Mann-Whitney U test and LASSO algorithm to perform dimensionality reduction and feature selection on radiomic features. The aim was to reduce redundant features and optimize the fitting model. In the initial stage of feature selection, the Mann-Whitney U test was applied to two independent samples to identify radiomic features with *P*<0.05, which were then used as candidate features for further in-depth analysis using the LASSO algorithm. During the implementation of the LASSO algorithm, 200 random seeds were set, and the optimal tuning parameter (λ) was determined through 5-fold cross-validation. As log(λ) varied from -10 to -1, the number of features included in the model gradually decreased, and the absolute values of the variable coefficients approached 0, allowing the algorithm to select and determine the final optimized variables progressively.

Based on the results of the LASSO regression, a radiomic model, referred to as Rad-score, was constructed. Univariate (*P*<0.2) combined with multivariate logistic stepwise regression (*p* < 0.05) was employed to select the optimal traditional PET metabolic parameters and clinical factors for constructing a clinical model. A combined model was created by integrating Rad-score, traditional PET metabolic parameters, and clinical factors.

ROC curve analysis was planned, and AUC was calculated to evaluate the predictive ability of the clinical model, Rad-score, and combined model for EGFR gene mutations. Calibration curves were utilized to assess the robustness of each model. The DeLong test was conducted to determine whether there were performance differences among the models. Finally, the clinical utility of the best model in predicting EGFR mutations in lung adenocarcinoma was evaluated through a clinical decision analysis curve.

In the training set, a nomogram was developed based on the Rad-score and clinical variables. This graphical representation serves as a straightforward and user-friendly clinical tool that effectively displays the prediction results for each patient.

### 2.7 Statistical Methods

Python 3.9.7 and IBM SPSS 26.0 software were used for statistical analysis. Continuous variables that conformed to a normal distribution were expressed as *x̄* ± *s* s, while those that did not conform to a normal distribution were expressed as M (P25, P75). Qualitative data were presented as frequency (percentage). Comparisons between the two groups were performed using the independent-samples t-test or the Mann–Whitney U test, and comparisons of categorical variables were conducted using the χ^2^ test. ROC curves were generated for the prediction results of each model, and the AUC, sensitivity, specificity, accuracy, and F1-score were calculated. The DeLong test was used to compare differences in the performance of each model. A *p*-value < 0.05 was considered statistically significant.

## 3 RESULTS

### 3.1 Analysis of Clinical Data and PET Metabolic Parameters

A total of 93 patients with pathologically confirmed lung adenocarcinoma were enrolled in this study. Based on EGFR genetic testing results, the cohort included 63 patients (67.7%) in the mutation group and 30 patients (32.3%) in the wild-type group. This distribution aligns with the reported prevalence of EGFR mutations in Asian populations [3, 24, 25]. Among the 63 patients with EGFR mutations, exon 19 deletions (19-del) were the most common subtype (n = 28, 44.4%), followed by L858R point mutations in exon 21 (n = 22, 34.9%). Other less common mutations, including those in exon 20 (*e.g*., T790M, S768I) and exon 18 (*e.g*., G719X), accounted for 13 cases (20.6%). Due to the limited sample size within each uncommon mutation subgroup, subsequent radiomic analyses were conducted based on overall mutation status (mutant *vs*. wild-type) rather than individual subtypes to ensure statistical robustness and avoid model overfitting.

All patients were randomly divided into a training set of 47 cases (31 mutation cases and 16 wild-type cases) and a testing set of 46 cases (32 mutation cases and 14 wild-type cases) at a 1:1 ratio . There were no significant differences in clinical data (gender, age, smoking history, tumor maximum diameter, TNM stage) or traditional PET metabolic parameters (SUVmax, SUVmin, SUVmean, SUVpeak, SULmax, SULmin, SULmean, SULpeak, TLG) between the mutation group and wild group in both the training and testing sets (all *P*>0.05). Then, to determine significant clinical factors and traditional PET metabolic parameters for modeling, univariate logistic regression analysis was used for screening, but no statistically significant clinical variables or PET metabolic parameters were found (*P*>0.05) (Table **1**).

To avoid missing potential clinical risk factors and related PET metabolic parameters, variables with *P*<0.2 in univariate analysis were retained, including gender (*P*=0.188) and smoking history (*P*=0.062). These variables were included in a multivariate logistic stepwise backward regression for further screening. The results showed that non-smokers were more prone to EGFR mutations, and there was a significant difference in smoking history between the mutation group and the wild group (*P*<0.05).

### 3.2 Feature Selection and Radiomics Model/Rad-score Construction

**Fig. (1):**
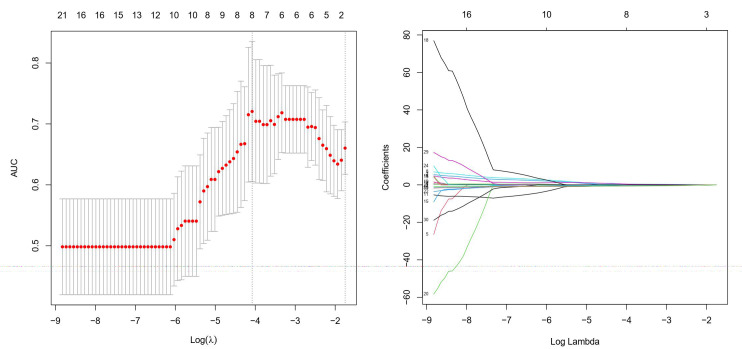
Optimal subset of radiomic features extracted using LASSO algorithm and 5-fold cross-validation ROC curves of each model on the test set.

A total of 1781 PET radiomic features and 1781 CT radiomic features were extracted from each patient. First, the Mann-Whitney U test was used to screen the radiomic features with *P* < 0.05, resulting in a total of 453 features. These features were then used as candidate features for LASSO regression analysis. During LASSO regression analysis, the model achieved optimal predictive performance at log(λ) = -4 (Fig. **1**). This value was used to select the most relevant features, resulting in eight radiomic features with non-zero coefficients.

6 features were based on PET images

(1) wavelet-HLH_GLCM_Correlation_PET,

(2) wavelet-HLH_firstorder_Kurtosis_PET,

(3) wavelet-HLL_GLSZM_SmallAreaHighGrayLevelEmp hasis_PET,

(4) wavelet-LHL_GLSZM_SmallAreaLowGrayLevelEmp hasis_PET,

(5) wavelet-LHL_firstorder_Range_PET,

(6) wavelet-LLH_firstorder_Kurtosis_PET;

2 features were based on CT images

(1) log-sigma-5-0-mm-3D_firstorder_Skewness_CT,

(2) wavelet-HHL_firstorder_Mean_CT.

**Fig. (2):**
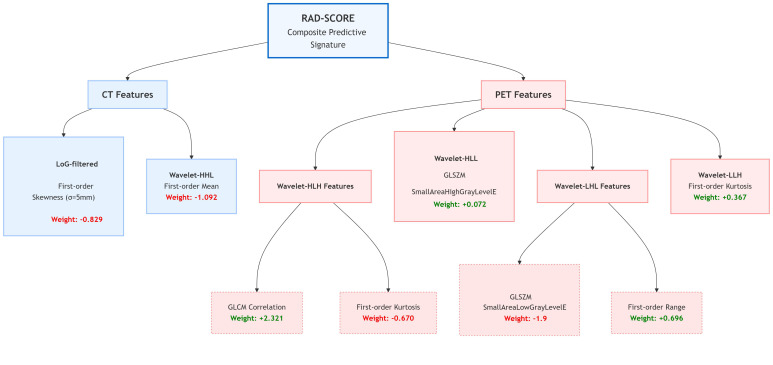
Rad-score formula structure decomposition. **Abbreviations: GLSZM:** gray level size zone matrix; **GLCM:** gray level co-occurrence matrix.

An imaging radiomic model for predicting EGFR gene mutations in lung adenocarcinoma was constructed based on the weights of these features, *i.e*., a formula was established to calculate the Rad-score for each patient (Fig. **2**).

### 3.3 Construction and Validation of Clinical Model and Combined Model

Eight radiomic features, one clinical variable (smoking history), and their combination were used to construct three models: radiomics model, clinical model, and combined model. The ROC curves for these models on the training and test sets are shown in Figs. (**3** and **4**). The AUC, sensitivity, specificity, accuracy, and F1-Score results are presented in Table **2**.

In the training set, the combined model demonstrated the highest predictive accuracy, as reflected by its largest AUC. When compared to the clinical model, both the combined model and the radiomics model achieved significantly greater accuracy and specificity, not only in the training set but also in the testing set. In the testing set, the radiomics model demonstrated not only a satisfactory AUC but also the highest F1-score, indicating a balanced trade-off between its ability to correctly identify mutated cases and its overall predictive accuracy for a positive result.

The DeLong test results showed that there were significant differences in AUC values between the radiomics model, combined model, and clinical model in the training set (*P*=0.0173; *P*=0.002), while there was no significant difference in the testing set (*P*=0.428; *P*=0.594). The combined model combining radiomics features and clinical factors showed slightly improved performance in predicting EGFR mutations in lung adenocarcinoma compared to the radiomics model, with AUC values of 0.885 (95% CI, 0.785-0.985) and 0.714 (95% CI, 0.542-0.886) in the training and testing sets, respectively.

According to the Delong test results, there was no significant difference in AUC values between the radiomics model and the combined model in both the training and testing sets (*P*=0.284; *P*=0.371). The clinical model had the poorest diagnostic performance in both the training and testing sets. In the training set, there were no significant differences in sensitivity, specificity, and accuracy between the radiomics model and the combined model. However, in the testing set, the radiomics model had better sensitivity, while the combined model had better specificity.

### 3.4 Construction and Validation of the Nomogram

A personalized prediction nomogram was developed by integrating the clinically significant variable (smoking history) that exhibited significant differences in the multivariate logistic regression analysis, along with the Rad-score, as illustrated in Fig. (**5**). This nomogram effectively presents prediction results and the relative weights of each factor. The calibration curves for the three models are depicted in Fig. (**6**), which assesses their stability.

### 3.5 Clinical Application

**Fig. (7):**
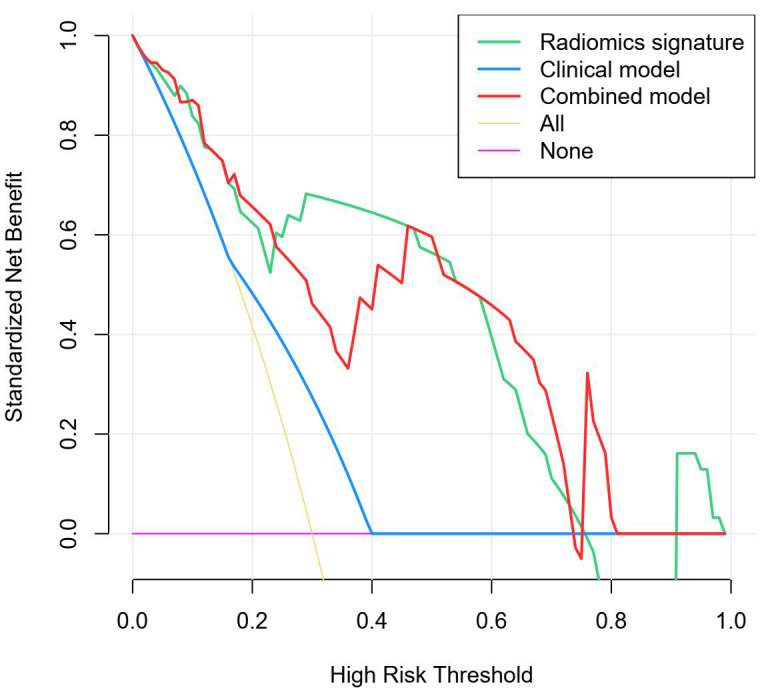
Decision curves of three models on the training set. **All:** Represents performing clinical intervention for all patients. **None:** Represents not performing clinical intervention for any patients.

**Fig. (8):**
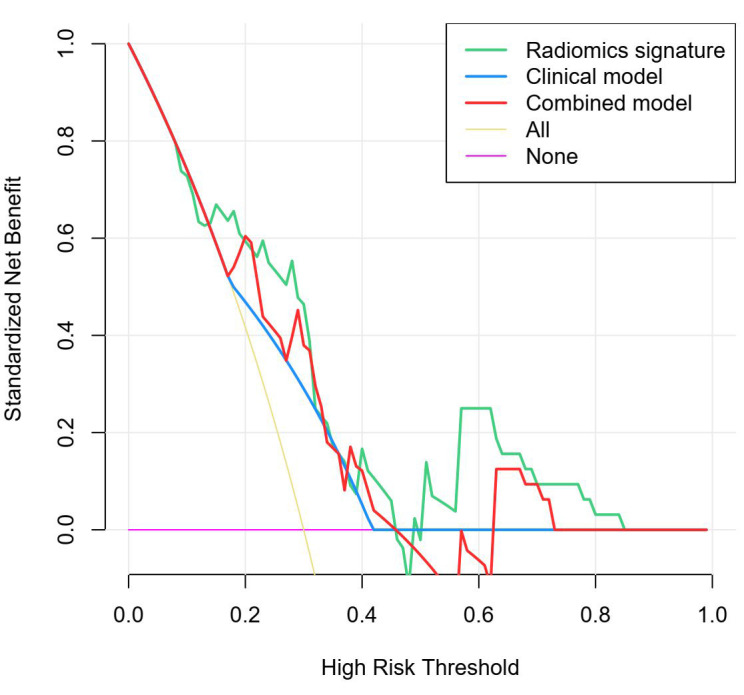
Decision curves of three models on the testing set. **All:** Represents performing clinical intervention for all patients. **None:** Represents not performing clinical intervention for any patients.

Decision curve analysis (DCA) illustrates the net benefit rate across various risk thresholds (Figs. **7** and **8**). The horizontal axis represents the threshold probability; when the predicted probability exceeds a specific threshold, the model classifies the patient as having a mutation, and the corresponding clinical treatment is administered. The vertical axis reflects the net benefit, representing the advantages patients gain from accurate predictions and effective clinical management. Therefore, a higher net benefit indicates greater clinical application value of the model. Analysis of the decision curves for both the training and testing datasets shows that, across a broad range of threshold probabilities, both the combined model and the radiomics model demonstrate higher net benefits compared with the single clinical model.

## 4 DISCUSSION

^18^F-FDG PET/CT is widely used in the diagnosis, staging, treatment response assessment, and prognosis prediction of lung adenocarcinoma. For instance, Lee *et al*. [26] demonstrated that bone marrow uptake on PET/CT was an independent prognostic factor in small cell lung cancer, while another study by Lee *et al*. [27] showed dual-time-point imaging improved the prediction of distant metastasis in NSCLC.

While traditional treatments primarily involved chemotherapy and radiotherapy, targeted therapies and immunotherapies-particularly for advanced inoperable patients-have now become mainstream. EGFR is the most common driver gene in lung adenocarcinoma, and EGFR-TKIs have been shown to significantly prolong progression-free survival in mutation-positive patients, as evidenced by Zhu *et al*.'s comprehensive review of clinical outcomes [28].

Currently, tissue biopsy remains the gold standard for detecting EGFR mutations. However, due to factors such as patient refusal, procedural risks, tumor location, or intratumoral heterogeneity-demonstrated by Zhang *et al*. [29] through multi-region sequencing analysis-obtaining sufficient tissue for genetic testing is not always feasible. Therefore, developing a non-invasive method to predict EGFR status is of great clinical importance.

The most commonly used PET parameter, SUVmax, has yielded inconsistent results across studies. Lim *et al*. [30] reported lower SUVmax in EGFR-mutant tumors, while Kanmaz *et al*. [31] found higher SUVmax values associated with mutations. These discrepancies may stem from differences in patient cohorts, imaging protocols, or technical factors. In our study, EGFR-mutant tumors tended to show lower SUVmax, consistent with Lim *et al*. [30], but the difference was not statistically significant.

Given the limitations of SUVmax, volumetric parameters such as MTV and TLG have been introduced. Minamimoto *et al*. [32] reported that quantitative PET metrics could predict EGFR mutation with AUC values up to 0.77, while Lee *et al*. [33] identified TLG as an independent prognostic factor. However, results remain variable across studies [32-35], and in our cohort, TLG was not an independent predictor of EGFR mutation, similar to findings by Whi *et al*. [34]. These collective findings suggest that conventional metabolic parameters alone are insufficient for reliably predicting EGFR status, highlighting the need for more advanced approaches such as radiomics.

Radiomics provides a promising solution by extracting high-dimensional, quantifiable features from medical images. Although many radiomics studies have focused on CT or MRI, ^18^F-FDG PET/CT offers unique advantages by capturing both metabolic and morphological information. Zuo *et al*. [36] developed a multi-center PET/CT radiomics model that achieved an AUC of 0.76 in predicting EGFR status, while a recent meta-analysis by Chen *et al*. [37] confirmed that machine learning approaches could achieve a pooled sensitivity of 0.78 and specificity of 0.80 in mutation prediction. Chen *et al*. [37] noted in their systematic review that PET-based models generally outperformed CT-only approaches in genetic characterization.

Our findings are consistent with, yet extend, the work of Ning Ma, who also reported that a combined PET/CT radiomics model outperformed models based on either modality alone in predicting EGFR mutations [38]. However, our study diverges in a crucial aspect: we explicitly tested the added value of integrating clinical factors with radiomic features. Contrary to our initial hypothesis, the combined model (Rad-score + smoking history) did not yield a statistically significant improvement in AUC over the radiomics-alone model. This suggests that the selected radiomic features may already encapsulate the biological manifestations of the mutational phenotype, making the addition of basic clinical variables redundant for this specific prediction task. This finding is critical, as it implies that a well-developed imaging biomarker could potentially stand alone, simplifying its potential clinical translation.

However, previous studies have paid less attention to clinical factors. To improve the predictive ability of PET/CT imaging for EGFR mutation status in lung adenocarcinoma, we constructed prediction models based on PET/CT radiomics and incorporated clinical factors to evaluate their predictive efficacy and clinical feasibility. The EGFR mutation rate in this study's patient population was 67.7% (63/93), similar to the previously reported mutation rate in Asian populations (36.8%-76.2%) [24]. In the training set, in addition to radiomics features, we considered including 14 other variables, such as clinical factors and traditional PET metabolic parameters. However, through univariate and multivariate logistic regression variable selection, we found that only smoking history was associated with EGFR mutations, with non-smokers being more prone to EGFR mutations. Therefore, smoking history was ultimately included in the construction of the clinical model. Previous studies have shown that EGFR mutations are relatively more common in female, non-smoking, and Asian lung adenocarcinoma patients [39], and tumors carrying EGFR mutations are usually shorter in length and smaller in volume compared to EGFR wild-type tumors [20]. However, this study did not find gender or tumor maximum diameter to be independent predictors of EGFR mutations.

Ultimately, this study utilized eight PET/CT radiomics features (six from PET and two from CT images) to construct a Rad-score, established a clinical model based on smoking history, and developed a combined model integrating both factors. The AUC values consistently demonstrated the superiority of imaging-based models: radiomics model (training: 0.865; testing: 0.737), clinical model (training: 0.637; testing: 0.645), and combined model (training: 0.885; testing: 0.714). These findings align with previous radiomics studies [20, 36] while providing deeper insight into feature selection-specifically, the dominance of PET-derived features (75% of selected features) underscores the critical role of metabolic heterogeneity in characterizing EGFR-driven tumors, consistent with the metabolic implications of EGFR signaling pathways reported by Huang *et al*. [11].

Notably, while the combined model achieved the highest training AUC, its performance gain over the radiomics model alone was not statistically significant in either training or testing sets (*P* > 0.05). This suggests that the radiomic features may have already encapsulated the biological manifestations of smoking-related carcinogenesis, limiting the additive value of clinical history. This observation contrasts with some previous studies [21, 40] that reported improved performance with clinical-radiomic integration, possibly due to differences in feature selection methodologies or cohort characteristics.

In the testing set, the radiomics model demonstrated higher sensitivity (71.4% *vs*. 64.3%), whereas the combined model showed superior specificity (84.4% *vs*. 81.2%). This nuanced performance profile echoes the findings of Zhang *et al*. [22], who similarly reported a trade-off between sensitivity and specificity in multi-feature models. The radiomics model also exhibited better calibration and higher net benefit in DCA across most probability thresholds, reinforcing its potential as an effective screening tool to minimize false negatives-a critical consideration in clinical practice where missing treatable mutations have significant consequences.

These results collectively indicate that while both radiomics and combined models effectively predicted EGFR status, the radiomics model demonstrated marginally better overall clinical utility. The limited improvement contributed by smoking history suggests that well-developed radiomic signatures may potentially operate as stand-alone predictors, simplifying clinical implementation. The comprehensive performance metrics revealed a nuanced profile for each model. The high sensitivity of the radiomics model is clinically desirable for a screening tool, as it minimizes the risk of missing candidates for targeted therapy. Conversely, the high specificity and precision of the combined model could be valuable in contexts where confirming a mutation with high certainty is prioritized, such as when considering costly therapies.

Our study has several limitations. First, the sample size, although comparable to previous single-center radiomics studies [19, 36], remains moderate. More importantly, the inherent class imbalance between the EGFR mutation group (n=63) and the wild-type group (n=30) may introduce bias and affect the generalizability of our predictive model. While we employed statistical techniques like LASSO regression, which are relatively robust to imbalance, and used AUC (area under the ROC curve) as a primary performance metric, which is less sensitive to class distribution than accuracy, future validation with a larger, more balanced cohort is essential.

Second, as noted in the results, we did not perform radiomic analysis for specific EGFR mutation subtypes (*e.g*., 19-del *vs*. L858R) due to the insufficient number of cases in each subgroup. Previous studies have suggested that different EGFR mutation subtypes may exhibit distinct biological behaviors and metabolic phenotypes [31]. Therefore, the radiomic features identified in our model represent a generalized signature for EGFR mutational status as a whole. A larger multicenter study is warranted to investigate whether radiomics can further discriminate between specific driver mutations, with profound implications for personalized treatment strategies.

Finally, only 14 clinical factors and PET metabolic parameters were used, resulting in insufficient breadth and depth of analysis. We plan to include additional clinical and imaging data to further explore and enhance the potential of the constructed model for predicting EGFR mutation efficacy.

## CONCLUSION

The radiomics model developed using ^18^F-FDG PET/CT imaging features effectively predicts EGFR gene mutations in patients with lung adenocarcinoma, providing valuable insights for guiding personalized precision treatment in clinical practice.

The combined model, which integrates smoking history with the radiomics model, demonstrates predictive efficacy for EGFR mutations in lung adenocarcinoma that is comparable to that of the radiomics model alone. However, an analysis of the calibration curve and clinical decision curve indicates that the radiomics model provides greater clinical value in evaluating EGFR mutation efficacy. Despite promising results, the model's performance should be further validated in larger, prospective, multi-center cohorts to address potential biases arising from sample size and class imbalance, and to assess its efficacy in predicting specific mutation subtypes.

## Figures and Tables

**Fig. (3):**
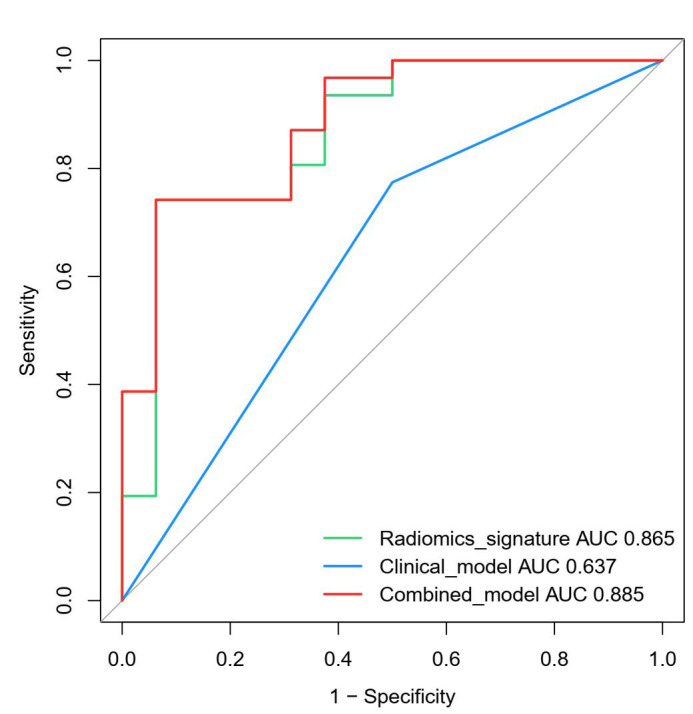
ROC curves of each model on the training set.

**Fig. (4):**
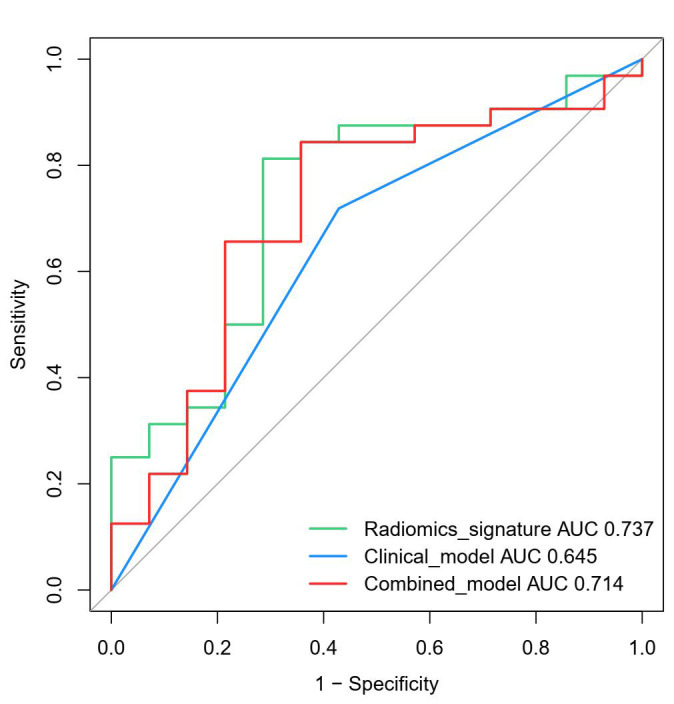
ROC curves of each model on the testing set.

**Fig. (5):**
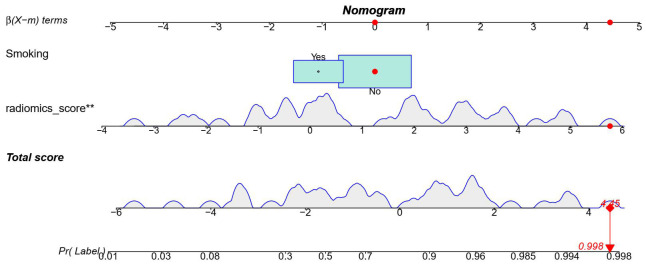
Nomogram based on the combined model.

**Fig. (6):**
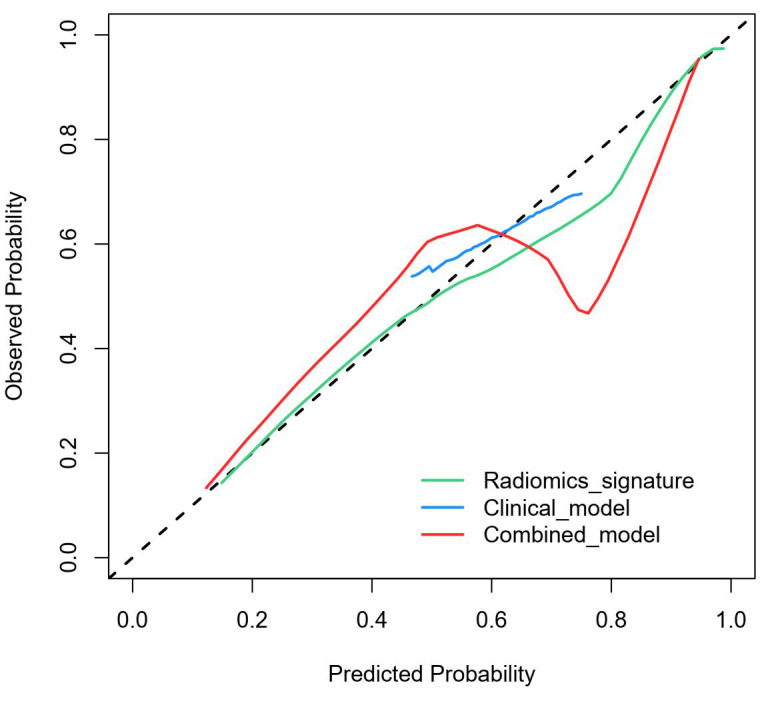
Calibration curves of three models on the training set.

**Table 1:** Comparison of clinical characteristics and pet metabolic parameters in lung adenocarcinoma patients with different egfr mutation statuses in the training set.

**Pathological Features**	**Mutation Group (n=31)**	**Wild-Type Group (n=16)**	**OR Value**	***p*-value**
Age (years; *x̄* ± *s*	63.94±9.61	67.31±14.29	0.972	0.337
Tumor Long Diameter (cm; *x̄* ± *s*)	3.86±1.96	3.33±1.76	1.176	0.369
Gender [n(%)]				
Male	15(47.8%)	11(68.8%)	2.347	0.188
Female	16(52.2%)	5(31.2%)		
Smoking[n(%)]				
Yes	7(22.6%)	8(50.0%)	3.429	0.062
No	24(77.4%)	8(50.0%)		
TNM Stage[n(%)]				
Non-Stage IV	17(54.8%)	7(43.8%)	1.561	0.472
Stage IV	14(45.2%)	9(56.2%)		
SUVmax[M(P_25_,P_75_)]	9.72(7.47,12.60)	9.08(5.30,12.72)	1.038	0.618
SUVmin[M(P_25_,P_75_)]	0.43(0.27,0.46)	0.43(0.33,0.54)	1.038	0.977
SUVavg[M(P_25_,P_75_)]	5.75(4.35,7.39)	5.31(3.17,7.02)	1.080	0.556
SUVpeak[M(P_25_,P_75_)]	7.93(5.30,11.13)	7.26(4.04,10.58)	1.055	0.546
SULmax[M(P_25_,P_75_)]	7.60(5.21,10.34)	7.30(3.93,10.58)	1.025	0.781
SULmin[M(P_25_,P_75_)]	0.33(0.21,0.36)	0.34(0.28,0.41)	0.839	0.917
SULavg[M(P_25_,P_75_)]	4.27(3.14,5.73)	4.27(2.34,5.84)	1.060	0.710
SULpeak[M(P_25_,P_75_)]	6.59(3.8,9.21)	5.84(3.09,8.67)	1.067	0.494
TLG[M(P_25_,P_75_)]	121.80(20.78,118.44)	79.66(7.33,138.18)	1.002	0.366

**SUVmax** = maximum standardized uptake value, **SUVmin** = minimum standardized uptake value,** SUVmean** = mean standardized uptake value, **SUVpeak** = peak standardized uptake value, **TLG** = total lesion glycolysis,** SULmax** = maximum standardized uptake value of lean body mass,** SULmin** = minimum standardized uptake value of lean body mass, **SULmean** = mean standardized uptake value of lean body mass, **SULpeak** = peak standardized uptake value of lean body mass.

**Table 2:** Diagnostic performance of prediction model in training and testing datasets.

**Model**	**Set**	**AUC (95% CI)**	**Accuracy**	**Sensitivity**	**Specificity**	**Precision**	**F1-Score**
**Radiomics Model**	Training	0.865 (0.747-0.983)	80.9%	74.2%	93.8%	95.2%	83.3%
	Testing	0.737 (0.572-0.901)	78.3%	71.4%	81.2%	83.3%	76.9%
**Clinical Model**	Training	0.637 (0.490-0.784)	68.1%	50.0%	77.4%	69.2%	58.3%
	Testing	0.645 (0.489-0.801)	67.4%	57.1%	71.9%	76.2%	65.3%
**Combined Model**	Training	0.885 (0.785-0.985)	80.9%	74.2%	93.8%	95.2%	83.3%
	Testing	0.714 (0.542-0.886)	78.3%	64.3%	84.4%	85.7%	73.7%

**AUC:**Area Under the Receiver Operating Characteristic Curve, measures the overall model performance. **F1-Score:**The harmonic mean of Precision and Sensitivity, providing a single metric that balances both concerns. A higher F1-score indicates a more robust and balanced model. The radiomics model demonstrated the best balanced performance with the highest F1-score in the test set. The combined model showed the highest specificity, which is valuable for confidently ruling out wild-type cases.

## Data Availability

The data of the current study are available from the corresponding author [S.F], upon a reasonable request.

## LIST OF ABBREVIATIONS

NSCLC: Non-small-cell lung cancer
EGFR: Epidermal growth factor receptor
TKIs: Tyrosine kinase inhibitors
^18^F-FDG: ^18^F-fluorodeoxyglucose
PET/CT: Positron Emission Tomography / Computed Tomography
OSEM: Ordered subsets expectation maximization
SUV: Standardized uptake value
MTV: Metabolic tumor volume
TLG: Total lesion glycolysis
AUC: Area under the curve
LASSO: Least Absolute Shrinkage and Selection Operator
SVM: Support Vector Machine
SUL: Standardized uptake value of lean body mass
SUVmax: Maximum standardized uptake value
SUVmin: Minimum standardized uptake value
SUVpeak: Peak of standardized uptake value
SULmax: Maximum standardized uptake value of lean body mass
SULmin: Minimum standardized uptake value of lean body mass
SULmean: Mean standardized uptake value of lean body mass
SULpeak: Peak of standardized uptake value of lean body mass
ROI: Region of interest
VOI: Volume of interest
GLCM: Gray level co-occurrence matrix
GLRLM: Gray level run length matrix
GLSZM: Gray level size zone matrix
NGTDM: Neighboring gray tone difference matrix
GLDM: Gray level dependence matrix
WT: Wavelet transform
DCA: Decision curve analysis

## STANDARDS OF REPORTING

STROBE guidelines were followed.
